# One-step ionic liquid-based ultrasound-assisted dispersive liquid–liquid microextraction coupled with high-performance liquid chromatography for the determination of pyrethroids in traditional Chinese medicine oral liquid preparations

**DOI:** 10.1186/s13065-019-0578-7

**Published:** 2019-05-10

**Authors:** Yiyi Liao, Yuge Hou, Yan Zhong, Hong Chen, Chang Xu, Makoto Tsunoda, Yingxia Zhang, Shiming Deng, Yanting Song

**Affiliations:** 1 Key Laboratory of Tropical Biological Resources of Ministry of Education; Department of Pharmaceutical Sciences, School of Life and Pharmaceutical Sciences, Hainan University, Haikou, 570228 China; 2 Graduate School of Pharmaceutical Sciences, University of Tokyo, Tokyo, 113-0033 Japan

**Keywords:** Trace analysis, Dispersive solvent, Traditional Chinese medicine, Pyrethroids, Ultrasound

## Abstract

In this study, a simple one-step ionic liquid-based ultrasound-assisted dispersive liquid–liquid microextraction technique was coupled with high-performance liquid chromatography for the analysis of four pyrethroids in three kinds of traditional Chinese medicine oral liquid preparations: simotang oral liquid, kangbingdu oral liquid, and huaji oral liquid. The extraction parameters were examined to improve extraction efficiency. The optimum extraction conditions were 50 μL of 1-octyl-3-methylimidazolium hexafluorophosphate utilized as the extraction solvent and 800 μL of acetonitrile applied as the dispersive solvent. The extraction was assisted by ultrasonication for 8 min. The limits of detection for the four pyrethroids were within 0.007–0.024 mg L^−1^, and the limits of quantitation ranged between 0.023 and 0.080 mg L^−1^. The accuracy of the pyrethroid determination ranged from 80.1 to 106.4%. It was indicated that the proposed ionic liquid-based ultrasound-assisted dispersive liquid–liquid microextraction method had an easy operation and was accurate and environmentally friendly. This approach has potential for the analysis of pyrethroids in traditional Chinese medicine oral liquid preparations.

## Introduction

Traditional Chinese medicine (TCM) is widely employed in the treatment of a variety of diseases, including cough, hyperlipidemia, hypertension and infectious diseases [1, 2]. During the cultivation of Chinese herbal medicine, pesticides are commonly used to control pests and diseases. Currently, synthetic pyrethroid insecticides are more frequently used than traditional organophosphate [3], organonitrogen [4], organochlorine [5], and carbamate pesticides [6] because of their strong insecticidal activity and good stability upon exposure to light and air [7]. However, numerous studies have indicated that these pyrethroid pesticides are toxic to the nervous, reproductive, immune and cardiovascular systems [8]. Oral liquid is one of the most commonly used TCM preparations, and residues of pyrethroid pesticides in TCM oral liquid preparations greatly affect the patients’ health and course of therapy. Because TCM preparation contains a great many of herbal components, the interference of complex matrix to the pyrethroid residues and the limitation of current analytical methods will make the residue analysis of pyrethroid pesticides very difficult. Therefore, an accurate measurement method for the pyrethroid pesticides in TCM oral liquid preparations is urgently required.

Varieties of methods have been exploited for the measurement of pyrethroid residues, and the potential analytical methods include gas chromatography with electron capture detection [9] or mass spectrometry (MS) [10] and high-performance liquid chromatography (HPLC) with ultraviolet (UV) detection [11], diode array detection [12], or MS [13]. MS significantly improved the analysis of pyrethroid residues owing to its high sensitivity; however, it has stricter instrumentation requirements and is not suitable for some typical analytical laboratories. Among these techniques, HPLC–UV has been frequently employed in the analysis of pyrethroid residues [14–16]. However, the analysis of pyrethroid residues in TCM oral liquid preparations is difficult because of the extremely low pyrethroid concentrations and the complexity of the TCM sample. Therefore, pretreatment of the sample before HPLC analysis is crucial for the whole analysis process. Several approaches have been employed for the extraction of pyrethroids from samples, and these methods include the Soxhlet extraction [17], ultrasonic extraction [18], liquid–liquid extraction [19], and solid-phase extraction [20]. However, the extraction approaches have certain limitations, including large organic solvent consumption and a time-consuming extraction procedure.

Recently, ionic liquids (ILs)—semi-organic molten salts with an organic or inorganic anion and an organic cation—have emerged as alternative extraction solvents for sample treatment because of their advantages of strong thermal stability, good miscibility with organic and aqueous solvents, low vapor pressure, and good solubility for both organic and inorganic compounds. ILs have been utilized for the analysis of several kinds of organic compounds, such as benzoylurea insecticides, neonicotinoid insecticides, and endocrine-disrupting compounds [21–23]. Compared with conventional extraction methods, less organic solvent was consumed during IL dispersive liquid–liquid microextraction, and a higher extraction efficiency was achieved within a shorter extraction time.

The current study was performed to exploit a one-step ionic liquid dispersive liquid–liquid microextraction (IL-DLLME) for the sensitive measurement of the pyrethroid insecticide in TCM oral liquid preparations. In the current research, ultrasound technology was utilized to cause the ILs to disperse into the aqueous phase as well as to enrich the efficiency. The extraction conditions were examined to improve extraction efficiency. The current approach was then employed in the trace measurement of four pyrethroid insecticides in TCM oral liquid preparations.

## Methods

### Reagents and materials

**Fig. 1:**
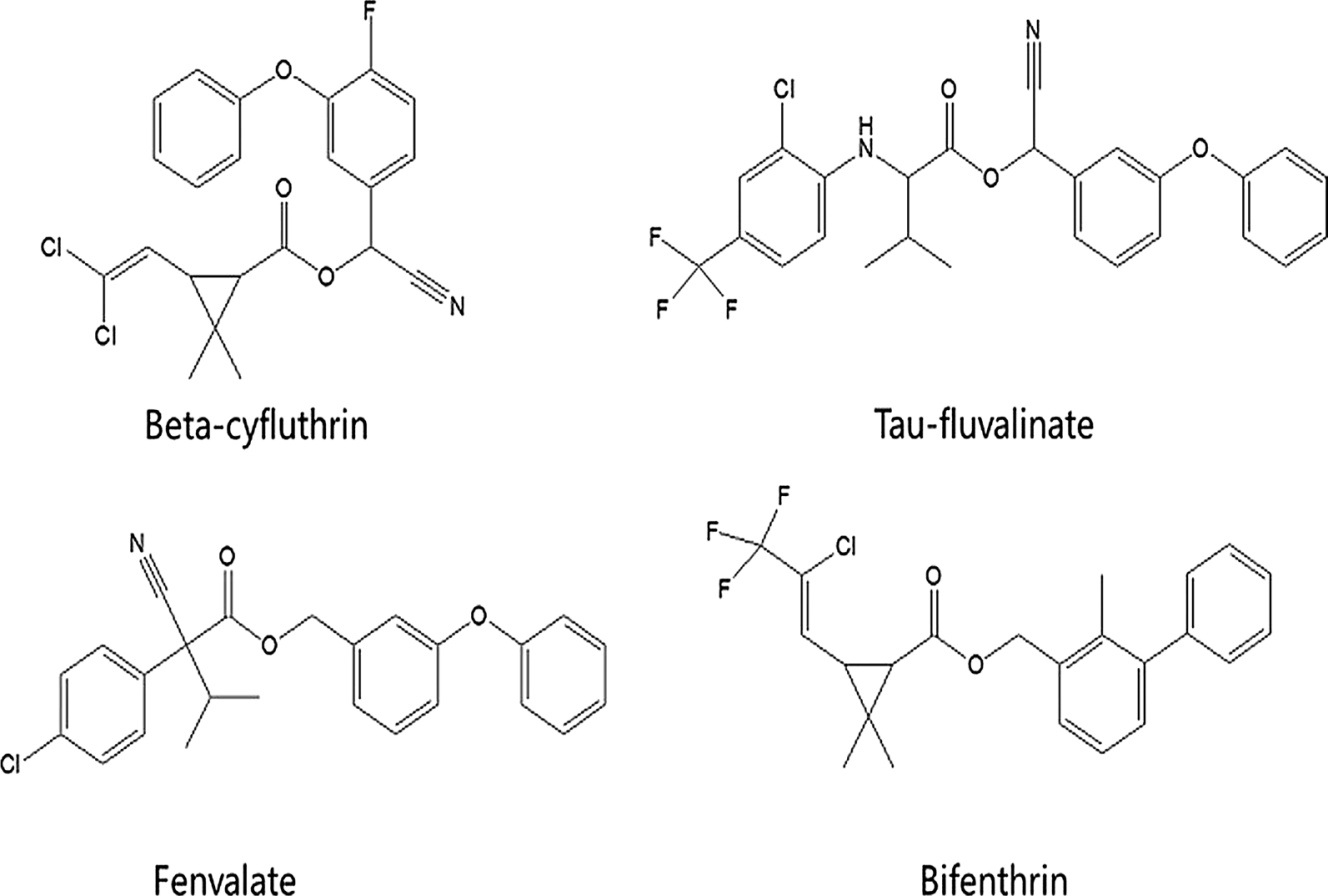
Chemical structures of four pyrethroids

Four pyrethroids (beta-cyfluthrin, bifenthrin, tau-fluvalinate and fenvalerate, Fig. 1) were purchased from Dr. Ehrenstorfer GmbH (Germany). The ILs 1-butyl-3-methylimidazolium hexafluorophosphate ([C_4_MIM][PF_6_]), 1-hexyl-3-methylimidazolium hexafluorophosphate ([C_6_MIM][PF_6_]), and 1-octyl-3-methylimidazolium hexafluorophosphate ([C_8_MIM][PF_6_]) were provided by the Lanzhou Institute of Chemical Physics, Chinese Academy of Sciences (Gansu, China). Acetonitrile (HPLC grade) was supplied by Mreda Corporation (USA). Water from a Milli-Q system (MA, USA) was employed after purification step. Three pyrethroid-free TCM oral liquid preparations were investigated in this study. Simotang oral liquid (composed of *Aucklandia lappa* Decne, *Citrus aurantium* L., *Areca catechu* L., and *Lindera aggregata* (Sims) Koster) was provided by Hansen Pharmaceutical Co., Ltd. (YiYang, China). Kangbingdu oral liquid (composed of *Isatis indigotica* Fort, *Phragmites communis* Trin, *Curcuma zvenyujin* Y. H. Chen et C. Ling, *Anemarrhena asphodeloides* Bge, *Acortw tatarinowii* Schott, *Pogostemon cablin* (Blanco) Benth, and *Forsythia suspensa* (Thunb.) Vahl) was obtained from Topsun Youbang Pharmaceutical Co., Ltd. (Huainan, China). Huaji oral liquid (composed of *Poria cocos* (Schw.) Wolf, *Sepia esculenta* Hoyle, *Callus gallus domesticus* Brisson, *Sparganium stoloniferum* Buch-Ham, *Curcuma phaeocaulis* Val, *Carthamus tinctorius* L., *Areca catechu* L., *Omphalia lapidescens* Schroet, *Carpesium abrotanoides* L., and *Quisqualis indica* L.) was provided by Chengzhi Pharmaceutical Co., Ltd. (Yongfeng, China).

### Apparatus

A KQ2200DE ultrasonic generator provided by Kunshan Ultrasonic Instruments Co., Ltd. (Jiangsu, China) was operated with an output power and frequency of 100 W and 40 kHz, respectively. An AXTGL16M desktop high-speed refrigerated centrifuge was purchased from Anxin Technologies Inc. (Jiangsu, China).

### Chromatographic conditions

The determination of pyrethroids was implemented on an HPLC system (Waters Corporation, USA) with a 1525 HPLC pump and a 2489 UV/visible detector. A Diamonsil C18 column (5 μm, 4.6 mm id × 150 mm) from Dikma Technologies Inc. (Beijing, China) was used. Eluent A was water/acetonitrile (95/5, v/v), and eluent B was water/acetonitrile (5/95, v/v). The mobile phases were eluted according to the following program: 12% (A) from 0 to 9.0 min, followed by 12–0% (A) from 9.0 to 35.0 min. The flow rate was 0.6 mL min^−1^, and the column temperature was 30 °C. The detection was monitored at 210 nm.

#### Optimization of IL-DLLME extraction method

The effects of extraction conditions, including the type of IL, IL volume, type of dispersive solvent, dispersive solvent amount, and ultrasonic extraction time, on the recoveries were optimized by single-factor experiments. The experiments were all carried out in triplicate.

#### IL-DLLME procedure

The TCM oral liquid preparations were centrifuged at 8000 rpm for 30 min, and the supernatant was filtered by a membrane filter (0.22 μm) before the IL-DLLME procedure. Then, 50 μL of [C_8_MIM][PF_6_] and 800 μL of acetonitrile were measured by microsampler and pipette respectively, and added to 5 mL of the filtered sample solutions in a conical tube. The ultrasound-assisted extraction (output power of 100 W and frequency of 40 kHz) was carried out for 8 min. Subsequently, 8 mL of the sample was centrifuged at 6153×*g* for 5 min. The pyrethroids were extracted into a droplet of IL settled at the bottom of the tube. A syringe was used to remove the upper aqueous phase. The IL phase containing the analytes was diluted with 70 μL of acetonitrile. Ultimately, 10 μL of the resultant solution was delivered into the chromatographic system for analysis.

#### Calculations

The enrichment factor (EF), defined as the ratio of the final concentration in the sediment phase (C_fin_) to the initial target component concentration in the TCM oral liquid preparation (C_ini_), was calculated as:

$$ {\text{EF}} = \frac{{{\text{C}}_{\text{fin}} }}{{{\text{C}}_{\text{ini}} }} $$

The extraction recovery (ER), which was utilized to estimate the pretreatment procedure under various experimental conditions, was calculated as follows:

$$ {\text{ER}} = \frac{{{\text{C}}_{\text{fin}} \times {\text{V}}_{\text{fin}} }}{{{\text{C}}_{\text{ini}} \times {\text{V}}_{\text{ini}} }} \times 100\% $$

where V_fin_ is the final target component concentration in the sediment phase and V_ini_ is the initial target component concentration in the TCM oral liquid preparation [23].

#### Preparation of spiked samples

Spiked samples were prepared by spiking appropriate amount of the standard solutions in the TCM oral liquid preparations to yield final concentrations of 20, 50 and 100 μg L^−1^ for four pyrethroids, respectively. Then the samples were subsequently prepared according to the upper IL-DLLME procedure.

## Results and discussion

### Type of IL

**Fig. 2:**
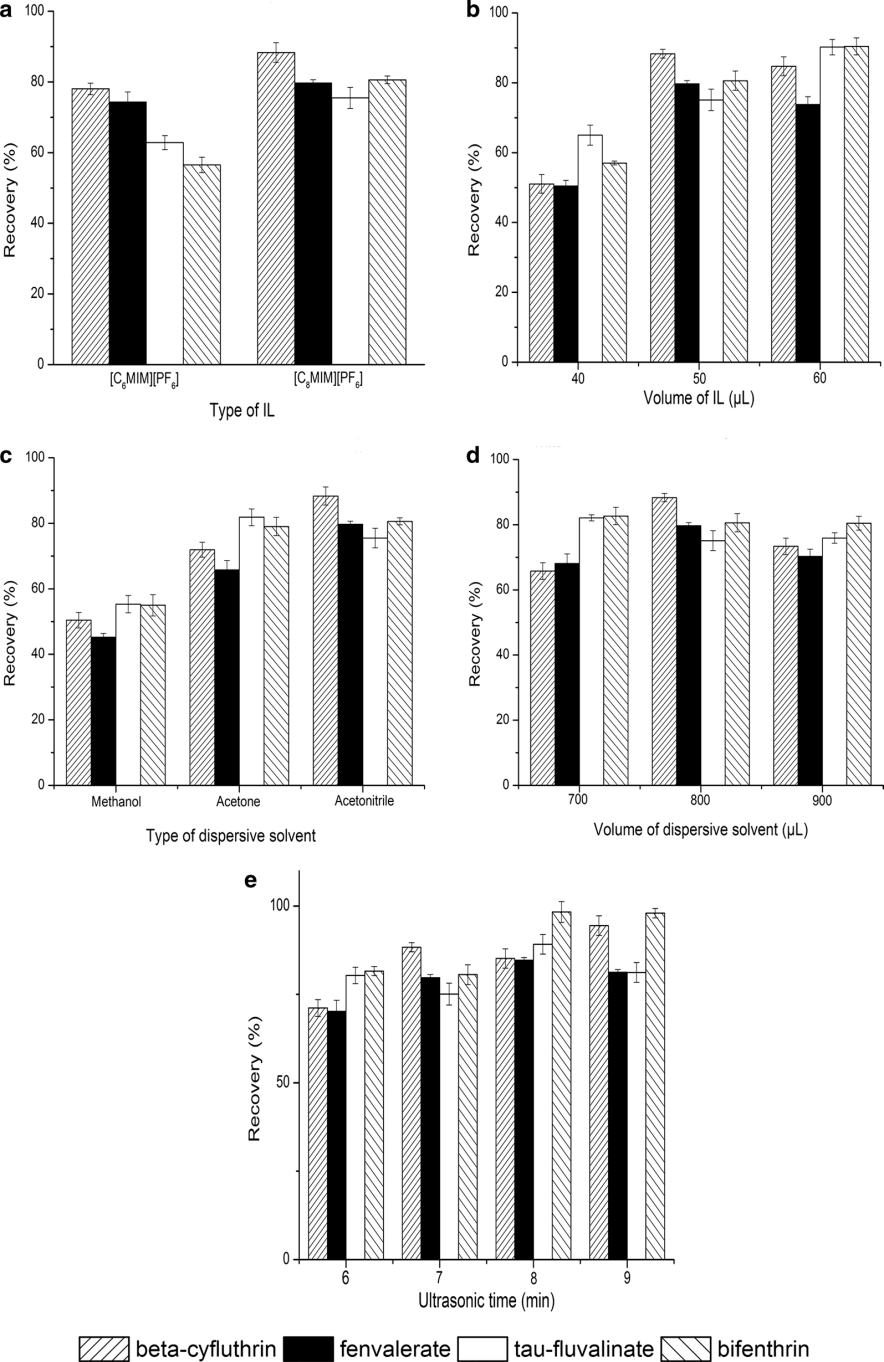
Effect of IL type (**a**), IL volume (**b**), dispersive solvent type (**c**), dispersive solvent volume (**d**) and ultrasonic extraction time (**e**) on the extraction recovery

The proper extraction solvent is vital for the success of the IL-DLLME process. The proper extraction solvent should have several key characteristics, including good chromatographic behavior, higher density than water, and lower water solubility [22, 24]. Three hydrophobic ILs ([C_4_MIM][PF_6_], [C_6_MIM][PF_6_] and [C_8_MIM][PF_6_]) were studied for IL-UA-DLLME in this study. However, the cloudy phase of [C_4_MIM][PF_6_] was difficult to form in the IL-DLLME process; therefore, [C_6_MIM][PF_6_] and [C_8_MIM][PF_6_] were compared for the extraction of four pyrethroid pesticides from TCM oral liquid preparations in the subsequent experiments. As presented in Fig. 2a, [C_8_MIM][PF_6_] obtained higher extraction recoveries than those of [C_6_MIM][PF_6_]. The longer alkyl chain may decrease the water solubility of IL, which contributes to higher extraction recoveries. Thus, [C_8_MIM][PF_6_] was applied as the extraction solvent during this research.

#### IL volume

When a smaller amount of IL was used, a small amount of precipitation formed, which indicated that the target compound could not be extracted efficiently and that repeatability was poor. In contrast, excess IL may decrease the enrichment factor and sensitivity of the analytical method. The optimum extraction volume of [C_8_MIM][PF_6_] was examined by the comparison of three different volumes (40 μL, 50 μL, and 60 μL). It could be observed that higher recoveries were obtained when 50 μL and 60 μL of [C_8_MIM][PF_6_] were used (Fig. 2b). Considering the enrichment factor and sensitivity of the analytical method, a lower volume of IL was preferred, and 50 μL of [C_8_MIM][PF_6_] was employed during the extraction.

### Type of dispersive solvent

To reduce interfacial tension and increase surface area between the two phases, a proper dispersive solvent with excellent miscibility during the one-step ionic liquid-based ultrasound-assisted dispersive liquid–liquid microextraction (IL-UA-DLLME) process is necessary. Methanol, acetonitrile and acetone were investigated (Fig. 2c). A higher extraction efficiency was achieved when the dispersive solvent was acetonitrile. Thus, acetonitrile was applied for the dispersive solvent.

### Dispersive solvent amount

The effect of the acetonitrile amount was examined by varying the amount from 700 to 900 μL (Fig. 2d). The highest extraction recovery was achieved with 800 μL of acetonitrile. Therefore, 800 μL of acetonitrile was added during the extraction procedure.

#### Ultrasonic extraction time

The ultrasonic extraction time was investigated from 6 to 9 min (Fig. 2e). When the ultrasonic extraction time was changed from 6 to 8 min, the recovery increased. However, with further extension of the extraction time from 8 to 9 min, the extraction recovery did not greatly vary. As a consequence, 8 min was selected as the ultrasonic extraction time.

#### Analysis of real TCM oral liquid preparations

Pyrethroid-free TCM samples were employed as blanks for the analytical method validation. As shown in Table 1, the limits of detection (LODs, signal/noise = 3) for the four pyrethroids were within 0.007–0.024 mg L^−1^, and the limits of quantitations (LOQs, signal/noise = 10) were within 0.023–0.080 mg L^−1^. The linearity was investigated within 0.1–10 mg L^−1^. The peak areas versus the injection amount was plotted, and linear regression equations were obtained. The correlation coefficients of the four pyrethroids were greater than 0.999.

**Table 1:** Analytical characteristics of the IL-DLLME method combined with HPLC–UV analysis

Samples	Analytes	Linearity equation	R^2^	Linear range (mg L^−1^)	LOD (mg L^−1^)	LOQ (mg L^−1^)	Enrichment factor	Extraction recovery (%)	Precision (% RSD)	
									Intra-day (*n *= 5)	Inter-day (*n *= 5)
Simotang oral liquid	Beta-cyfluthrin	y = 124,226.43x + 7577.64	0.9999	0.1–10	0.019	0.067	99	82.8	0.8	0.7
	Fenvalerate	y = 114418x + 7708.3	0.9999	0.1–10	0.024	0.080	98	92.5	1.4	2.5
	Tau-fluvalinate	y = 144216x + 9161	0.9999	0.1–10	0.011	0.037	103	91.2	3.0	3.5
	Bifenthrin	y = 167,084.12x + 8753.71	0.9999	0.1–10	0.010	0.033	114	91.8	0.8	1.2
Kangbingdu oral liquid	Beta-cyfluthrin	y = 111083x + 7169.3	0.9993	0.1–10	0.012	0.040	120	96.1	1.3	3.1
	Fenvalerate	y = 106583x + 5899.6	0.9993	0.1–10	0.016	0.053	112	89.2	2.9	2.1
	Tau-fluvalinate	y = 138510x + 16,352	0.9991	0.1–10	0.008	0.027	102	81.1	2.0	0.9
	Bifenthrin	y = 168772x + 7222	0.9999	0.1–10	0.007	0.023	102	81.0	1.7	2.3
Huaji oral liquid	Beta-cyfluthrin	y = 121491x + 16,306	0.9998	0.1–10	0.019	0.067	131	85.1	1.2	3.1
	Fenvalerate	y = 114327x + 5899.6	0.9999	0.1–10	0.021	0.070	129	83.8	2.9	3.9
	Tau-fluvalinate	y = 140657x − 14,435	0.9999	0.1–10	0.009	0.030	136	88.3	2.0	2.2
	Bifenthrin	y = 158771x + 14,879	0.9997	0.1–10	0.011	0.036	150	97.7	1.7	1.4

As presented in Table 1, the relative standard deviation (RSD) values were 0.8–2.9% for the intra-day precision and 0.7–3.9% for the inter-day precision. The pyrethroids in spiked samples were determined at three concentrations (20, 50 and 100 μg L^−1^). As shown in Table 2, the average recoveries were within 80.1–106.4%.

**Table 2:** Analysis of the TCM oral liquid preparations and spiked recoveries (n = 3)

Samples	Spiked level (μg L^−1^)	Relative recovery ± RSD (%)			
		Beta-cyfluthrin	Fenvalerate	Tau-fluvalinate	Bifenthrin
Simotang oral liquid	20	95.7 ± 1.3	86.8 ± 2.8	100.6 ± 2.9	103.0 ± 1.2
	50	83.7 ± 2.6	84.8 ± 2.5	89.7 ± 2.9	98.6 ± 2.6
	100	92.7 ± 2.1	90.8 ± 2.5	94.6 ± 1.6	106.0 ± 0.8
Kangbingdu oral liquid	20	94.7 ± 0.9	82.8 ± 2.9	91.0 ± 2.6	91.2 ± 2.1
	50	96.1 ± 2.1	89.2 ± 2.7	81.1 ± 1.4	81.0 ± 2.2
	100	97.2 ± 2.4	89.5 ± 2.4	90.2 ± 2.7	106.4 ± 1.1
Huaji oral liquid	20	81.9 ± 3.0	81.5 ± 1.1	81.5 ± 1.3	94.7 ± 2.2
	50	85.1 ± 3.1	83.8 ± 3.6	88.3 ± 2.2	97.7 ± 5.0
	100	84.4 ± 2.1	83.4 ± 2.3	80.1 ± 1.3	94.2 ± 2.7

**Fig. 3:**
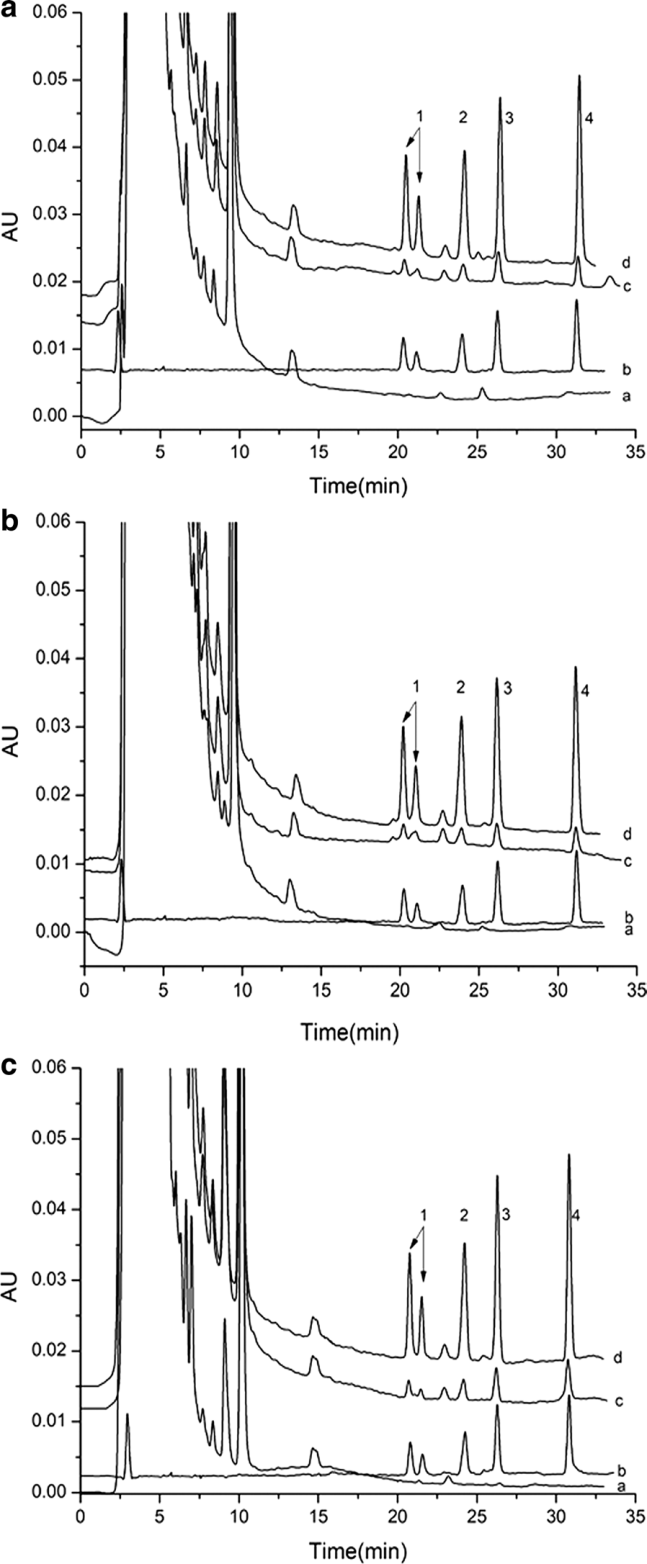
Typical chromatograms of four pyrethroids in oral liquids—**a** simotang oral liquid, **b** kangbingdu oral liquid, **c** huaji oral liquid—using optimum conditions: (1) beta-cyfluthrin, (2) fenvalerate, (3) tau-fluvalinate, and (4) bifenthrin. In chromatograms (**a**, **c**, **d**), the spiked levels were 0, 20, 100 μg L^−1^, and **b** shows the standard solution

Three kinds of TCM oral liquid preparations (simotang, kangbingdu, and huaji) were obtained from a local community pharmacy and were analyzed with HPLC–UV after the IL-UA-DLLME procedure. A typical high-performance liquid chromatogram under the conditions described in "Chromatographic conditions" section is shown in Fig. 3. The pyrethroids were separated successfully in the spiked oral liquid (0, 20 and 100 μg L^−1^) and spiked blank (50 μg L^−1^), indicating that the components in the TCM oral liquid preparations did not interfere with the analysis of the pyrethroids. Thus, the IL-DMLLE pretreatment approach is applicable for the measurement of pyrethroids in TCM oral liquid preparations.

### Comparison of the present approach with other approaches

Table 3 lists a comparison of the current analytical approach with the published approaches for the measurement of pyrethroids in liquid samples [25–28]. Compared with other techniques, the current method consumed fewer types and lower amounts of organic solvents. In addition, although the sample matrix of a TCM oral liquid is very complex, the proposed method can provide LODs and enrichment factors comparable to other methods. Because this method has no special instrumentation requirements, it is highly applicable for the routine analysis of pyrethroids in TCM oral liquid preparations.

**Table 3:** Comparison of IL-UA-DLLME with other methods for the determination of pyrethroids in liquid samples

Sample	Analyte	Extraction method	Detection method	Extraction solvent	Organic solvent consumption (mL)	Sample volume (mL)	LOD	Linear range	Recovery (%)	EF	Refs.
Fruit juices	Tetramethrin, fenpropathrin, cypermethrin, deltamethrin, fenvalerate, permethrin	DLLME^a^	HPLC–UV	Chloroform	1.25 mL of methanol, 0.3 mL of chloroform	5.00	2.0–5.0 μg L^−1^	2.0–1500 μg L^−1^	84–94	62–84	[25]
Water	Ethofenprox, lambda-cyhalothrin, d-phenothrin, bifenthrin	IL-DLLME^b^	HPLC–UV	[C_6_MIM][PF_6_]	0.6 mL of methanol	5.00	10.38–15.56 μg L^−1^	50–2000 μg L^−1^	88–98	260–319	[26]
Water	Allethrin, cypermethrin, prallethrin, tetramethrin, transfluthrin, and imiprothrin	UA-DLLME^c^	HPLC–UV	Tetrachloromethane	20 μL of tetrachloromethane, 1.0 mL of acetone	10.00	0.1–0.3 μg L^−1^	0.6–1520 μg L^−1^	86–109	767–1033	[27]
Vegetable oils	Fenpropathrin, sumithrin, cyhalothrin, permethrin, deltamethrin	LLE-DLLME^d^	GC-FID^e^	Dimethylformamide	4.5 mL *n*-hexane, 1 mL DMF,	5.00	0.02–0.17 mg kg^−1^	0.06–6 mg kg^−1^	85–109	40–70	[28]
TCM oral liquid	Beta-cyfluthrin, bifenthrin, tau-fluvalinate, fenvalerate	UA-DLLME	HPLC–UV	[C_8_MIM][PF_6_]	0.8 mL of acetonitrile	5	7–24 μg L^−1^	0.1–10 mg L^−1^	80.1–106.4	98–150	This work

^a^Dispersive liquid–liquid microextraction

^b^Ionic liquid dispersive liquid–liquid microextraction

^c^Ultrasound-assisted dispersive liquid–liquid microextraction

^d^Liquid–liquid extraction-dispersive solid-phase extraction

^e^Gas chromatography-flame ionization detector

## Conclusions

In the current work, a sensitive analytical approach was investigated for the measurement of four pyrethroids in TCM oral liquid preparations by the utilization of IL-UA-DLLME coupled with HPLC. The extraction parameters were investigated to improve the extraction efficiency, and excellent enrichment performance was achieved. The chromatographic conditions were also tested, and the chromatographic determination was achieved within 35 min. Compared with previous studies, although the sample matrix is more complex on account of the various of herbal component in TCM oral liquid, the proposed method achieved similar LODs with the utilization of less types and lower volume of toxic organic solvents during the microextraction procedure. The results reveal that the method is an accurate, simple, and environmentally friendly method for analyzing the pyrethroids in TCM oral liquid preparations.

## Abbreviations

[C_4_MIM][PF_6_]: 1-butyl-3-methylimidazolium hexafluorophosphate
[C_6_MIM][PF_6_]: 1-hexyl-3-methylimidazolium hexafluorophosphate
[C_8_MIM][PF_6_]: 1-octyl-3-methylimidazolium hexafluorophosphate
EF: enrichment factor
ER: extraction recovery
IL-DLLME: ionic liquid dispersive liquid–liquid microextraction
ILs: ionic liquids
IL-UA-DLLME: ionic liquid-based ultrasound-assisted dispersive liquid–liquid microextraction
TCM: traditional Chinese medicine
